# DNA Methylation in Atherosclerosis: A New Perspective

**DOI:** 10.1155/2021/6623657

**Published:** 2021-06-23

**Authors:** Yan Zhang, Jun Mei, Jing Li, Ying Zhang, Qingbing Zhou, Fengqin Xu

**Affiliations:** ^1^Xiyuan Hospital, China Academy of Chinese Medical Sciences, Beijing 100091, China; ^2^Institute of Geriatric Medicine, Xiyuan Hospital, China Academy of Chinese Medical Sciences, Beijing 100091, China

## Abstract

Atherosclerotic cardiovascular diseases, in which atherosclerosis (AS) is the main pathologic basis, are currently the primary diseases leading to human deaths. Emerging evidence showed that DNA methylation, which could affect the transcription and expression of critical regulatory genes, has key roles in AS. Aberrant DNA methylation including aberrant hypomethylation and hypermethylation plays key roles in endothelial-cell dysfunction, macrophage inflammation, abnormal proliferation of vascular smooth muscle cells, plaque rupture, and thrombosis in AS. Chinese herbal medicines, including single compounds and formulations, showed light on the treatment of AS through regulating the aberrant DNA methylation in AS. Targeting the aberrant DNA methylation may be one of the most important treatment strategies in the cure and prevention of AS. In this review, we focus on the relationship between DNA methylation and AS, as well as the beneficial effects of Chinese herbal medicines on DNA methylation in AS.

## 1. Introduction

Atherosclerosis (AS) includes coronary heart disease and stroke, which are the leading causes of death worldwide [1]. The pathologic mechanism of AS mainly involves injury to the vascular intima caused by dyslipidemia; foam cell (FC) formation; proliferation of vascular smooth muscle caused by inflammatory mediators and plaque rupture [2–4]. Treatments for AS include lipid-lowering drugs, anti-inflammatory agents, and antiplatelet aggregation, but a cure is lacking.

DNA methylation induces changes in gene expression without changing the DNA sequence by adding methyl groups to cytosine in a CpG-containing nucleotide to form 5-methylcytosine5 [5]. Thanks to the development of technology for detecting DNA methylation, the relationship between DNA methylation and the occurrence and development of AS has been clarified. Some scholars believe that AS is an epigenetic disorder [6, 7]. Emerging studies have shown that abnormal methylation of DNA plays a very important part in the inflammatory response, endothelial injury, FC formation, and smooth muscle cell (SMC) proliferation [8–10]. Thus, it is thought that DNA methylation is strongly associated with the occurrence and development of AS. Targeting such aberrant methylation of DNA in AS is an important strategy for the prevention and treatment of AS and has attracted the attention of many researchers.

DNA methyltransferase inhibitors (DNMTIs) are US Food and Drug Administration-approved agents for the treatment of hematologic malignancies and include 5-aza-2′-deoxycytidine (decitabine) and 5-azacytosine (azacytidine). Interestingly, DNMTIs have been found to also have a beneficial effect on AS-related diseases [11, 12]. However, they have not been used against AS in clinical trials.

As reported by the World Health Organization, 80% of people in developing countries use herbal medicines (HMs) for healthcare. Some studies [13, 14] have revealed that single compounds and herbal formulations can regulate DNA methylation in AS. Considering the extensive application of HMs and their potential role in the regulation of DNA methylation, further research is needed.

In this review, we focus on recent findings of the role of DNA methylation in AS. We also discuss the regulatory effects of HM on DNA methylation in AS treatment.

## 2. Abnormal Methylation of DNA in AS

Studies have shown that aberrant DNA methylation including aberrant hypermethylation and hypomethylation plays an important in AS [15]. In healthy individuals, 5′-C-phosphate-G-3′ (CpG) islands in the promoter region of genes are, in general, hypomethylated, whereas CpG islands in the nonpromoter region are hypermethylated [16]. Global DNA hypomethylation (which is known as DNA hypomethylation of nonpromoter regions) can cause structural changes and instability of chromosomes because of the initiation of transcription at incorrect regions and high transcriptional activity in sites that are usually silent. Global DNA hypomethylation leads to expression of potentially harmful genes and also high expression of genes that are meant to be silent. Conversely, global DNA hypermethylation causes inactivation of disease-suppressor genes or protective genes, gene mutation, and allelic loss. In total, DNA methylation is catalyzed by DNA methylation transferases (DNMT1, DNMT3A, and DNMT3B) and reversed by TET proteins (TET1, TET2, and TET3) [17, 18].

### 2.1. Aberrant Hypomethylation of DNA in AS

Evidence linking AS with DNA hypomethylation has been found in many studies in humans and animals [19]. Aberrant hypomethylation of the genome has been found in advanced human atherosclerotic lesions, apolipoprotein defect (ApoE^−/−^) mice with atherosclerotic plaques, and SMC in New Zealand white rabbits [20]. Castro et al. found that patients with vascular disease had a significantly low global level of DNA methylation. Lund et al. assessed the levels of DNA methylation in the early stages of AS and discovered that DNA hypomethylation was present in the aorta and peripheral blood monocytes from ApoE^−/−^ mice [21]. Einari et al. measured the methylation status in femoral atherosclerotic plaques from 22 cases on a genome-wide basis. They found that there were many abnormal hypomethylated sites in the promoter region of the AS genome, accompanied by relatively few abnormal hypermethylated sites (3997 versus 782), when compared with those in nine normal cases [22]. Another study demonstrated that hyperhomocysteinemia and other risk factors such as aging and consumption of a high-fat diet could lead to a decline in the methylation level of genomic DNA [23–25]. Taken together, these results suggested that aberrant hypomethylation is associated with AS development.

### 2.2. Aberrant Hypermethylation of DNA in AS

In recent years, the number of reports on the occurrence and development of AS caused by DNA hypermethylation has been increasing. Studies have shown that the hypermethylation of Kruppel-like factor (KLF) 2 is observed in AS, which inhibits KLF2 expression [26]. Similarly, the hypermethylation of KLF4 and cellular repressor of E1A-stimulated genes (CREG) were induced by the increasing of DNMT3a and DNMT3b [10, 27]. Aberrant hypermethylation of KLF2, KLF4, and CREG can reduce mRNA expression, which eventually increases inflammation and causes endothelial dysfunction in vascular endothelial cells.

Taking aberrant hypomethylation into account, these results suggest that the balance of DNA methylation in AS is disturbed. Aberrant hypermethylation and hypomethylation both have an effect on AS development, which indicates that not only the aberrant hypermethylation but also the aberrant hypomethylation could be the potential targets for the AS treatment.

## 3. DNA Methylation Is Correlated with the Pathologic Mechanism of AS

**Figure 1:**
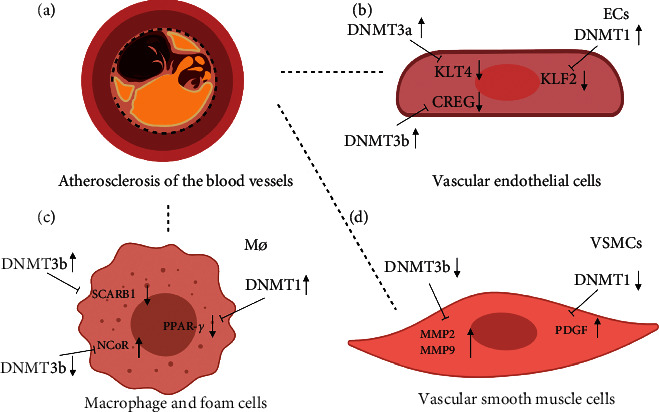
Cell type-specific changes in DNA methylation in atherosclerosis. (a) Atherosclerosis of blood vessels. (b) In vascular endothelial cells, disturbed blood flow and ox-LDL can upregulate expression of DNA methyltransferases, which then reduce expression of KLF4, KLF2, and CREG. (c) In macrophages and foam cells, upregulated expression of DNA methyltransferases can decrease SCARB1 and PPAR‐γ expression; downregulated expression of DNMT3b can increase expression of NCoR. (d) In smooth muscle cells, downregulated expression of DNMT3b/DNMT1 increases expression of MMP2, MMP9, and PDGF.

Several studies have demonstrated that DNA methylation is related to different stages of AS [9, 26, 28]. How DNA methylation triggers AS development is a crucial question. Four important pathologic mechanisms of AS have been postulated: (i) inflammation and endothelial dysfunction; (ii) macrophages and FC formation; (iii) proliferation of vascular smooth muscle cells (VSMCs); (iv) plaque rupture and thrombosis in AS (Figure 1).

### 3.1. Aberrant DNA Methylation in Inflammation and Endothelial Dysfunction

KLF2, KLF4, and CREG are protective factors with anti-AS effects. KLFs are a subtype of the zinc finger transcription factor family that have important roles in the differentiation and growth of cells [29]. KLF2 is an anti-inflammatory transcription factor that regulates fat differentiation [30]. Upregulated expression of DNMT1 causes methylation of the promoter region of KLF2 in human umbilical vein endothelial cells (HUVECs) treated with oxidized low-density lipoprotein (ox-LDL) [26]. Endothelial KLF4 is an important anti-inflammatory factor. *In vitro*, the increasing expression of DNMT3a increases the methylation level of the KLF4 promoter region and inhibits expression of KLF4 in HUVECs [10]. Similarly, upregulation of DNMT3b expression is accompanied by hypermethylation of CREG, which decreases the mRNA expression of CREG and aggravates endothelial dysfunction [27]. The three studies mentioned above also found that the DNMT inhibitor 5-aza-2′-deoxycytidine (5-Aza-dC) [10, 26, 27], RG108 [10], and an antioxidative molecule N-acetylcysteine (NAC) [27] can restore aberrant hypermethylation in endothelial cells through demethylation. However, these drugs have not entered the clinical stage and approved by the FDA for the treatment of patients with AS [11].

### 3.2. Aberrant DNA Methylation and Macrophages Derived FCs in AS

Macrophages phagocytose ox-LDL and turn into FCs, which form the earliest lipid streaks of AS. DNA methylation has been shown to modulate macrophages in AS. Homocysteine (Hcy) is an independent risk factor for AS and mediates lipid accumulation in AS. DNMT3b can accelerate Hcy-mediated AS by inhibiting the expression of scavenger receptor class B type 1 (SCARB1), which has been attributed to the reduced binding of specificity protein 1(SP1) to the SCARB1 promoter region [31]. MicroRNA (miR)-221 inhibits ox-LDL-induced macrophagic inflammation by inhibiting DNMT3b-mediated DNA methylation in the nuclear receptor corepressor (NCoR) promoter region [32]. Yu et al. established a model with macrophage-specific overexpression of DNMT1 (Tg^DNMT1^) or peroxisome proliferator-activated receptor (PPAR)−*γ* (Tg^PPAR−*γ*^) in ApoE^−/−^ mice. *In vitro* and *in vivo* experiments showed that Tg^DNMT1^ significantly increased the production of proinflammatory cytokines (e.g., tumor necrosis factor-*α* (TNF-*α*), interleukin- (IL-) 6, and IL-1*β*) in macrophages and plasma and accelerated AS progression in ApoE^−/−^ mice. PPAR-*γ* was found to be a target of DNMT1-regulated DNA methylation, and the increase in DNMT1 expression and decrease in PPAR-*γ* expression increased the inflammatory response [33]. 5-Aza-Dc may apply its AS-protective effects through antagonizing infiltration and activating immune cells (e.g., macrophages) induced by demethylation [12].

### 3.3. Aberrant DNA Methylation and VSMCs in AS

The migration and proliferation of arterial SMCs is a prominent feature of AS. Patients with advanced AS show significant hypomethylation of the genome of CpG islands in atherosclerotic plaques. Moreover, balloon denudation of the aorta of New Zealand white rabbits causes proliferation of intimal SMCs in which concomitant genomic hypomethylation is present, suggesting that genomic hypomethylation may have a role in SMC proliferation [20]. Matrix metalloproteinases (MMPs) and platelet-derived growth factor (PDGF) can promote the proliferation of atherosclerotic SMCs. Chen et al. found that miRNA-29b reduced the methylation level of MMP-2/MMP-9 by inhibiting DNMT3b expression, then increasing expression of MMP-2/MMP-9, and promoting the migration of human aortic smooth muscle cells [34]. Zhang et al. found that Hcy could increase the methylation level of PDGF in vascular endothelial cells by inhibiting the activity and expression of DNMT1, which promoted the proliferation and migration of VSMCs [35].

However, contrary results have also been reported. Ubiquitin-like PHD and RING finger domain-containing protein 1 (UHRF1) (as the apparent total regulator of VSMC plasticity) expression has been found to have a correlation between methylation and miRs. The key gene *miR-145* can control the translation of *Uhrf1* mRNA, and the plasticity of VSMCs is reduced. Meanwhile, DNA and histone methylation inhibits the promoter of cell cycle suppressor genes, which trigger VSMC proliferation. *In vivo* and *in vitro* experiments have suggested that *Uhrf1* is a potential therapeutic intervention target because it can normalize the transformation of the VSMC phenotype and reduce the vascular complications of AS [28].

Conversely, TET methylcytosine dioxygenase 2 (TET2) can modify DNA methylation by catalyzing 5-methylcytosine to 5-hydroxymethylcytosine and mediate DNA demethylation. TET2 is related to the phenotypic transformation of VSMCs, endothelial dysfunction, and inflammation of macrophages [36]. The underlying mechanisms are mostly associated with the methylation/demethylation regulation of related genes, such as myocardin, serum response factor [37], KLF4 [38], and autophagy-related genes [39]. Therefore, TET2 could provide a new target for the prevention and efficacious treatment of AS.

### 3.4. Aberrant DNA Methylation, Plaque Rupture, and Thrombosis in AS

The final stage of AS is the rupture of atherosclerotic plaques and thrombosis. Changes in hemodynamics activate platelets and a mural thrombus is formed. Activated platelets release cytokines, thereby promoting SMC proliferation and exacerbating formation of a plaque matrix. Rupture of atherosclerotic plaques and thrombosis can impede blood flow. MMPs and tissue factor pathway inhibitor 2 (TFPI-2) are connected to VSMC growth and plaque stability. TFPI-2 is a serine protease inhibitor. It can reduce thrombus formation by inactivating Xa and the factor TF-VII*α* complex and inhibiting MMPs to maintain the stability of atherosclerotic plaques [40]. Zawadzki et al. found that aberrant hypermethylation of TFPI-2 with low mRNA expression was present in carotid atherosclerotic plaques compared with those in a control group. They also discovered that the methylation level of TFPI-2 was higher in unstable plaques than that in stable plaques, suggesting that TFPI-2 hypermethylation was associated with vulnerable plaques in AS [41]. Platelet mitochondrial DNA (mtDNA) methylation is an emerging and innovative biomarker of cardiovascular disease [42]. Bordoni et al. study found that, after supplementation with L-carnitine for 6 months, the mtDNA methylation level of platelets was significantly increased, especially in the D-loop region. The result showed that L-carnitine protects the cardiovascular disease by regulating mtDNA [43].

## 4. The Anti-AS Effects of HMs *via* Regulation of DNA Methylation

Studies have shown that the balance of DNA methylation in AS is lost, which demonstrates the necessity for developing drugs for bidirectional regulation of DNA methylation in AS. Increasing numbers of studies have shown that Chinese HMs show a potential regulatory effect on DNA methylation in AS. Chinese HMs can (i) regulate the methylation level across the genome; (ii) regulate methylation of candidate genes; (iii) allow methylation to interact with miRs. Two classic components of HMs, single compounds and formulations, show potential clinical application targeting aberrant methylation of DNA in AS.

### 4.1. Herbs and Herbal Compounds

Curcumin [(1E,6E)-1,7-bis(4-hydroxy-3-methoxyphenyl)hepta-1,6-diene-3,5-dione)] is derived from the rhizomes of the East Indian plant *Curcuma longa*. The polyphenols from this plant have strong antiproliferative activity and prevent the development of cardiovascular diseases [44]. Curcumin can affect the site-specific methylation of the RNA18*S*5 promoter region. This action leads to a decline in rDNA transcription, which inhibits the proliferation of VSMCs and delays AS progression [45]. Molecular-docking studies between curcumin and DNMT1 have suggested that curcumin covalently blocks the catalytic sulfur salt of DNMT1 C1226 and has an inhibitory effect. In addition, curcumin has been shown to induce global DNA hypomethylation in a leukemia cell line [46].

Geniposide (methyl (1S,4aS,7aS)-7-(hydroxymethyl)-1-[(2S,3R,4S,5S,6R)-3,4,5-trihydroxy-6-(hydroxymethyl)oxan-2-yl]oxy-1,4a,5,7a-tetrahydrocyclopenta[c]pyran-4-carboxylate is derived from plants of the genus *Gardenia*. Geniposide is also found in *Hedyotis diffusa*, *Radix scrophulariae*, *Eucommia ulmoides*, and *Paederia scandens*. Studies have shown that geniposide has antiatherosclerotic effects and can regulate the abnormal hypermethylated and hypomethylated genes in RAW264.7-derived FCs, which are involved in various biological pathways related to AS. It has been postulated that gardenoside could be used to treat AS through the regulation of DNA methylation [47].

Resveratrol (5-[(E)-2-(4-hydroxyphenyl)ethenyl]benzene-1,3-diol) is derived from the Chinese herb *Polygonum cuspidatum* (Asian knotweed/Hu Zhang) and is also found in grapes and red wine. It exhibits a protective role against cardiovascular diseases. The phosphatase and tensin homology deleted on chromosome 10 (PTEN) is a bispecific protein and lipid phosphatase whose high expression can inhibit a variety of physiological and pathological processes in VSMCs [48]. Ma et al. found that resveratrol inhibited smooth muscle cell proliferation by inhibiting homocysteine-induced PTEN hypermethylation, and the related mechanism was related to DNMT1 low expression [49] (Table 1).

### 4.2. Herbal Formulations

#### 4.2.1. Genome-Wide Methylation

Kang found that Xuefu Zhuyu capsules, Siji Sanhuang capsules, and their combined application could stabilize AS plaques and decrease levels of DNA methylation in serum and expression of DNMTs in an AS model in mice [50]. Intervention using Zhizi Chuanxiong capsules (ZCC) *in vivo* revealed that ZCC could conduct bidirectional regulation of abnormal hypermethylated genes and hypomethylated genes to treat AS in New Zealand white rabbits [14]. In addition, other herbal formulations, such as Danhong injections and Danggui Shaoyao powders, can achieve an anti-AS effect by inhibiting DNMT1 expression in plaques [51, 52]. An *in vitro* study found that Pinggan Qianyang Fang could inhibit the proliferation and migration of VSMCs, which can lead to genomic DNA hypermethylation through adjustment of DNMT1 expression [53] (Table 2).

#### 4.2.2. Methylation of Candidate Genes

Chinese HM formulations comprise various Chinese medicines in different proportions. Recent studies have shown that several traditional Chinese medicine (TCM) formulations exert their biological effects on the occurrence and development of AS through a pathway of DNA methylation. Estrogen receptor (ER) *α* has been shown to protect against AS. The aberrant hypermethylation of ER*α* can reduce ER*α* expression, thereby leading to a higher risk of cardiovascular disease. In a model of postmenopausal AS mice and Hcy-treated HUVECs, Liuwei Dihuang pills were found to delay postmenopausal AS by increasing ER*α* mRNA and protein expression due to demethylation of ER*α* mediated by DNMT1 [13]. *In vivo* and *in vitro* studies have demonstrated that San Huang Xie Xin Tang increases expression of miRNA-152, inhibits DNMT1, and increases mRNA and protein expression of ER*α* [54]. The regulatory effect of TCM formulations on methylation of candidate genes has been shown in a KKAy mouse model. Shenqi compound can reduce the damage caused by the inflammatory response to vascular endothelial cells, protect vascular endothelial cells, inhibit SMC proliferation, and reduce the protein expression of TNF-*α*, toll-like receptor 4 (TLR-4), mammalian target of rapamycin (mTOR), and macrophage inflammatory protein 2 (MIP-2). The manner in which Shenqi compound protects diabetic macrovascular lesions may be related to modifications of aberrant hypermethylation of DNA involving mTOR, tumor necrosis factor receptor superfamily member 1B (Tnfrsf1b), peroxisome proliferator-activated receptor gamma coactivator 1-alpha (Ppargcl *α*), toll-like receptor 5 (Tlr5), arachidonate (12S)-lipoxygenase (Alox12), cytochrome P450 24A1 (CYP24A1), transcription factor 4 (TCF4), heparin-binding EGF-like growth factor (HB-EGF), and tumor necrosis factor-alpha-induced protein 8 (Tnfaip8) [55]. Danhong injections can achieve an anti-AS effect by reducing the methylation level of the autophagy-related gene autophagy-related protein 13 (Atg13) in the aorta of ApoE^−/−^ mice by inhibiting the phosphatidylinositol 3-kinase/Akt/mammalian target of rapamycin complex 1 (PI3K/Akt/mTORC1) signaling pathway [56]. Shen-Yuan-Dan capsules can decrease the methylation level of the Atg13 promoter region [57]. MMP-9 is a protease expressed mainly in macrophages, the main function of which is to degrade the extracellular matrix of the fiber cap (collagen, elastin, and casein) so as to make the atherosclerotic plaque unstable and rupture [58]. Li found that Soufeng Qutan Fang could reduce the methylation rate of ER and increase the methylation level of MMP9 in patients with acute coronary syndrome (ACS), suggesting that Soufeng Qutan Fang had a two-way regulatory effect on gene methylation [59]. The mechanism of action of Wen Ban Decoction combined with Western drugs in interventions of intermingled phlegm and blood stasis syndrome in ACS has been speculated to be related to the methylation status of the thrombomodulin gene promoter region, and the degree of methylation after treatment is significantly lower than before treatment [60]. Huang found that Yangxin Tongmai Formula affected methylation of zinc finger E-box-binding homeobox 2 (ZEB2) in coronary heart disease with blood stasis syndrome [61] (Table 3). The effects of Zhizi Chuanxiong capsule and Soufeng Qutan Fang on DNA methylation have been found to be bidirectional, which suggests that Chinese HMs could recover the balance of abnormal hypermethylation and hypomethylation of DNA in AS (Tables 2 and 3).

## 5. Conclusion and Perspectives

DNA methylation is an epigenetic modification that can play an important role in the control of gene expression. In general, hypermethylation typically suppresses expression, while hypomethylation leads to overexpression. Numerous studies have revealed that abnormal hypermethylation and hypomethylation of DNA both take part in the pathologic process of AS. Aberrant DNA methylation could affect the expression of AS-related genes and is expected to be a new target for AS treatment.

Emerging evidence indicates that finding effective therapeutic drugs from complementary and alternative medicine has been an important strategy in the cure of AS. It is worth noting that several studies have shown that herbal formulations and compounds can regulate the expression of mRNA or protein levels of AS-related genes by regulating DNA methylation (Tables 1–3), demonstrating that Chinese HMs could inhibit the development of atherosclerosis by regulating DNA methylation. Whether these protective effects will translate into better treatment effects for AS patients remains to be determined by clinical trials in the future.

## Figures and Tables

**Table 1:** Compounds from Chinese herbal medicines regulate DNA methylation in AS.

Herbs	Active compound	Target genes	Category of methylation effect	Mechanism of regulating DNA methylation	Models	Diseases	*In vitro/in vivo*	Reference
*Curcuma longa*	Curcumin	RNA18S5 and SIRT7	Hypermethylation	DNMT2↑	Vascular smooth muscle cells	AS	*In vitro*	[45]

*Gardenia* species	Geniposide	Akt2, Arrb, Tnf., and so on	Hypermethylation and hypomethylation		Ox-LDL-induced foam cells	AS	*In vitro*	[47]

*Polygonum cuspidatum*	Resveratrol	PTEN	Hypomethylation	DNMT1↓	Vascular smooth muscle cells	AS	*In vitro*	[49]

Sirtuin-7 (SIRT7), phosphatase and tensin homolog on chromosome 10 (PTEN), protein kinase Akt-2 (Akt2), arrestin beta 1 (Arrb), and tumor necrosis factor (Tnf).

**Table 2:** Herbal formulations regulate genome-wide methylation.

Formulation	Components	Category of methylation effect	Mechanism of regulating DNA methylation	Models	Diseases	*In vitro/in vivo*	Reference
Xuefuzhuyu capsule and Siji Sanhuang capsule	Dang Gui, Sheng Di Huang, Zhi Gan Cao, Tao Ren, Hong Hua, Zhi Qiao, Chi Shao, Chai Hu, Chuan Xiong, Jie Geng, Niu Xi, Da Huang, Huang Qin, Zhi Zi, and Huang Bai	Hypomethylation	DNMTs↓	ApoE^−/−^ mice fed with HFD	AS	*In vivo*	[50]

Zhizi Chuanxiong capsule	Zhi Zi and Chuan Xiong	Hypermethylation and hypomethylation		Rabbit fed with HFD	AS	*In vivo*	[14]

Danhong injection	Dan Shen and Hong Hua	Hypomethylation	DNMT1↓	ApoE^−/−^ mice fed with HFD	AS	*In vivo*	[51]

Danggui Shaoyao powder	Dang Gui, Chi Shao, Fu Ling, Bai Zhu, Ze Xie, and Chuan Xiong	Hypomethylation	DNMT1↓	ApoE^−/−^ mice fed with HFD	AS	*In vivo*	[52]

Pinggan Qianyang Fang	Tian Ma, Gou Teng, Shi Jue Ming, Mu Li, and Niu Xi	Hypermethylation	DNMT1↑	Angiotensin II-induced vascular smooth muscle cells	Vascular remodeling	*In vitro*	[53]

High-fat diet (HFD) and atherosclerosis (AS).

**Table 3:** Herbal formulations regulate the methylation status of AS-related genes.

Formulation	Components	Target genes	Category of methylation effect	Mechanism of regulating DNA methylation	Model	Diseases	*In vitro/in vivo*	Reference
Liuwei Ihuang pill	Shu Di Huang, Shan Zhu Yu, Fu Ling, Shan Yao, Mu Dan Pi, and Ze Xie	ER*α*	Hypomethylation	DNMT1↓	Hcy-induced apoptosis of human umbilical vein endothelial cells; postmenopausal AS model	AS	*In vitro/in vivo*	[13]

Sanhuang Xiexin Tang	Da Huang, Huang Qin, and Huang Lian	ER*α*	Hypomethylation	DNMT1↓	Lipopolysaccharide-treated human aortic smooth muscle cells; aorta from rats under a high-fat diet	AS	*In vitro/in vivo*	[54]

Shenqi compound	Sheng Dihuang, Huai Shanyao, Shan Zhuyu, Sheng Huangqi, Ren Shen, Dan Shen, Zhi Dahuang, and Tian Huafen	mTOR, Tnfrsf1b, Ppargcl*α*, Tlr5, Alox12, CYP24A1, TCF4, HB-EGF, and Tnfaip8	Hypermethylation		KKAy mice	Diabetes mellitus	*In vivo*	[55]

Dan Hong injection	Dan Shen and Hong Hua	Atg13	Hypomethylation		ApoE^−/−^ mice fed with HFD	AS	*In vivo*	[56]

Shen-Yuan-Dan capsule	Huang Qi, Dang Shen, Dan Shen, Di Long, Shui Zhi, Tu Yuan, Xuan Shen, and Yan Hu Suo	Atg13	Hypomethylation	DNMT1↓	ApoE^−/−^ mice fed with HFD	AS	*In vivo*	[57]

Soufeng Qutan Fang	Quan Xie, Wu Gong, Di Long, Chen Pi, Fa Ban Xia, Bai Zhu, Shui Zhi, and Jin Yin Hua	ER and MMP9	Hypermethylation and hypomethylation		Human peripheral blood cells	ACS	Clinical research	[59]

Wen Ban Tang	Quan Xie, Di Long, Chen Pi, Fa Ban Xia, Bai Zhu, Wu Gong, Shui Zhi, and Jin Yin Hua	Thrombomodulin	Hypomethylation		Human peripheral blood cells	ACS	Clinical research	[60]

Yangxin Tongmai Formulation	Ren Shen, Dan Shen, Zhi Shi, Fu Ling, and Gui Zhi	ZEB2	Hypermethylation		Human peripheral blood cells	Coronary heart disease	Clinical research	[61]

High-fat diet (HFD), atherosclerosis (AS), acute coronary syndrome (ACS), mammalian target of rapamycin (mTOR), tumor necrosis factor receptor superfamily member 1B (Tnfrsf1b), peroxisome proliferator-activated receptor gamma coactivator 1 alpha (Ppargcl *α*), toll-like receptor 5(Tlr5), arachidonate (12S)-lipoxygenase (Alox12), cytochrome P450 24A1 (CYP24A1), transcription factor 4 (TCF4), heparin-binding EGF-like growth factor (HB-EGF), tumor necrosis factor-alpha-induced protein 8 (Tnfaip8), autophagy-related protein 13 (Atg13), estrogen receptor (ER), matrix metalloproteinase-9 (MMP9), and zinc finger E-box-binding homeobox 2 (ZEB2).
